# Adverse childhood experiences and chronic pain in adults aged 86: findings from the Lothian Birth Cohort 1936

**DOI:** 10.3389/fragi.2025.1657525

**Published:** 2025-10-16

**Authors:** Dhaneesha N. S. Senaratne, Sam Singleton, Kate Timmins, Jeanette Spiteri, Paul Redmond, Adele Taylor, Janie Corley, Danielle Page, Janine Rennie, Huan Wang, Madeleine Verriotis, Suellen M. Walker, Debajit Sen, Gary J. Macfarlane, Lesley A. Colvin, Line Caes, Simon R. Cox, Tim G. Hales

**Affiliations:** ^1^ Chronic Pain Research Group, School of Medicine, University of Dundee, Dundee, United Kingdom; ^2^ Division of Population Health & Genomics, School of Medicine, University of Dundee, Dundee, United Kingdom; ^3^ Institute of Academic Anaesthesia, Division of Neuroscience, School of Medicine, University of Dundee, Dundee, United Kingdom; ^4^ Aberdeen Centre for Arthritis and Musculoskeletal Health (Epidemiology Group), School of Medicine, Medical Sciences and Nutrition, University of Aberdeen, Aberdeen, United Kingdom; ^5^ Lothian Birth Cohorts, Department of Psychology, University of Edinburgh, Edinburgh, United Kingdom; ^6^ Great Ormond Street Institute of Child Health, University College London, London, United Kingdom; ^7^ Department of Rheumatology, University College London Hospitals, NHS Foundation Trust, London, United Kingdom; ^8^ Division of Psychology, Faculty of Natural Sciences, University of Stirling, Stirling, United Kingdom

**Keywords:** chronic pain, childhood adversity, trauma-informed care, oldest adults, biopsychosocial model

## Abstract

**Background:**

Chronic pain, a major cause of disability, is prevalent in older people. Exposure to multiple adverse childhood experiences (ACEs) is associated with increased levels of chronic pain in later life. However, this association has not been investigated in people aged older than 80 years. Therefore, the primary objective of this study was to explore the relationship between ACEs and chronic pain in people with a mean age of 86 years, participants of the Lothian Birth Cohort 1936.

**Methods:**

A survey co-developed by researchers, clinicians and people with lived experience (PWLE) that assessed chronic pain and ACEs was completed by 229 participants (response rate 67%). Associations between ACE exposure and chronic pain were investigated using multinomial logistic regression.

**Results:**

Results showed that 58% reported chronic pain, with a higher prevalence in females. Furthermore, 69% of participants with chronic pain reported moderate or severe pain interference and 82% reported at least 1 ACE, with 25% indicating exposure to ≥4 ACEs. The most frequently reported exposure was community violence (48%). Males were more likely to report any ACE, physical neglect, bullying, and community violence. No significant association was found between ACEs and chronic pain status, severity, or interference in this cohort.

**Discussion:**

This study, the first to adapt ACE and chronic pain questionnaires with input from PWLE, suggests that the relationship between ACEs and chronic pain may be less relevant in people in their eighties compared to younger populations. These findings have implications for trauma-informed care and pharmacological treatment in older adults.

## Introduction

Chronic pain, defined as pain that persists or recurs for longer than 3 months, affects 18%–45% of adults worldwide and is one of the leading causes of years lived with disability (Fayaz et al., 2016; Sá et al., 2019; Zimmer et al., 2022; Ferrari et al., 2024; Rometsch et al., 2025). Several studies have reported that prevalence increases with age, though few exclusively explore populations over the age of 80 years (Fayaz et al., 2016; Li et al., 2021; LaRowe et al., 2024; Rometsch et al., 2025). As global populations continue to age, the number of people in this age category affected by chronic pain will continue to grow (United Nations, 2015).

There is increasing awareness that childhood circumstances can influence long-term health, including chronic pain (Felitti et al., 1998; Hughes et al., 2017; Bussières et al., 2023; Nicolson et al., 2023). Adverse childhood experiences (ACEs) are events or environments that have the potential to cause stress and undermine an individual’s sense of safety (CAPE, 2024). They include abuse (e.g., physical), neglect (e.g., emotional), household challenges (e.g., parental separation), or external challenges (e.g., bullying). An emerging hypothesis is that “toxic stress” experienced during development can alter biology and behaviour, leading to poor health outcomes in later life (Nelson et al., 2020). Neurobiological models highlight how repeated or severe early stress can produce long-term changes. The concept of allostatic load suggests that prolonged activation of the hypothalamic–pituitary–adrenal axis and autonomic nervous system leads to maladaptive changes, including immune dysregulation and neuroinflammation (McEwen, 2017; Santamaría-García et al., 2025). Childhood adversity has also been linked to alterations in brain structures implicated in pain perception (Antoniou et al., 2023). These changes may heighten vulnerability to central sensitization, an important contributor to chronic pain.

Additional perspectives extend beyond the toxic stress model in explaining the impact of childhood adversity on health throughout life. The field of life-course epidemiology posits that early adversity exerts lasting effects through the accumulation of risk across time (Kuh et al., 2003; Berens et al., 2017; Wagner et al., 2024). Childhood adversity may initiate a chain of events that influence educational attainment, socioeconomic circumstance, occupational exposures, and health risk behaviours, each of which could increase the occurrence of chronic pain in later life.

The biopsychosocial model of pain recognizes that disadvantage and psychological trauma shape pain outcomes (Engel, 1977; Uvelli et al., 2023). Early adversity is associated with insecure attachment styles and impaired emotion regulation, which can predispose to maladaptive coping strategies including heightened threat perception in connection with pain (Ciechanowski et al., 2003; Sheffler et al., 2025). Importantly, resilience factors such as strong social support and adaptive coping may buffer these risks, underscoring the heterogeneity of outcomes among individuals with ACE exposure.

Cumulative disadvantage theory suggests that the consequences of adversity compound over decades, shaping health trajectories into late life (Dannefer, 2003). In parallel, the inflammaging hypothesis proposes that older adults exhibit chronic low-grade inflammation, which may interact with immune dysregulation induced by early adversity to amplify pain risk (Franceschi and Campisi, 2014; Kuhlman et al., 2020). Conversely, it is also possible that associations between ACEs and pain attenuate in older populations due to survivor bias or age-related changes in stress reactivity.

These perspectives highlight several biological and psychosocial pathways through which childhood adversity may contribute to chronic pain across the life course, reinforcing the importance of investigating these associations in very old age.

The growing evidence for the role of ACEs in shaping health outcomes has prompted calls for trauma-informed practice, an approach to health and care interventions grounded in the understanding that trauma exposure can affect an individual’s neurological, biological, psychological, and social development (UK Government Office for Health Improvement and Disparities, 2022).

Whilst childhood stress can potentially be caused by a range of different events, many studies focus on a limited range of ACEs. For example, the widely used Childhood Trauma Questionnaire (CTQ) captures childhood maltreatment (i.e., abuse and neglect) but does not capture household or external challenges and so risks underestimating the burden of adversity (Bernstein et al., 2003; Nicolson et al., 2023; Mosler et al., 2025). Some instruments, such as the World Health Organization’s ACE International Questionnaire (ACE-IQ), report on a wider range of ACEs but are designed to be administered by a trained interviewer and are not suitable to be used as standalone instruments in a population cohort (World Health Organization WHO, 2018; Mosler et al., 2025).

Despite these limitations in assessing childhood circumstances, studies have consistently shown associations between ACEs and chronic pain in adolescents and adults, and recently inferred causal links (Groenewald et al., 2020; Bussières et al., 2023; Nicolson et al., 2023; Timmins et al., 2024). A recent meta-analysis including 80 studies revealed an odds ratio of 1.45 for developing chronic pain with any ACE exposure, rising to 1.95 after exposure to >4 ACEs (Bussières et al., 2023). Whether this connection is maintained in older populations is unclear, as one study with a mean participant age of 60 years demonstrated no association (Rouch et al., 2023).

In this study we sought to address the evidence gap by investigating exposure rates and associations between ACEs and chronic pain in a cohort of adults born in 1936, the Lothian Birth Cohort 1936 (LBC1936) (Deary et al., 2007), with an awareness that studying individuals aged greater than 80 years poses important challenges. Self-assessment of pain and adverse childhood experiences in very old age may be influenced by recall bias, sensory or cognitive impairment, generational differences in disclosure, or perception of adversity and pain. It is important to work with these challenges to overcome the absence of evidence regarding the impact of ACE exposure in this growing population. The mechanisms and observed associations may differ from those seen in younger populations.

We established the characteristics of chronic pain and ACEs and tested the primary hypothesis that self-reported exposures to ACEs were associated with an increase in chronic pain. We also examined the influence of sex and self-reported cognitive symptoms on reported exposure.

Females reported higher levels of chronic pain, while males reported greater exposure to ACEs and exposures were not affected by cognitive symptoms. Despite high rates of reporting childhood adversity and current chronic pain in both sexes, there were no significant associations observed in this population of 86-year-olds.

The findings imply that the oldest members of society, represented here by participants of LBC 1936, are resilient to the impact of ACEs on pain and therefore may not benefit from trauma-informed pain management strategies.

## Methods

This study is part of a broader multi-institution collaboration, the Consortium Against Pain Inequality (CAPE) (CAPE, 2024). CAPE consists of scientists, clinical researchers, pain specialists, epidemiologists, psychologists, and people with lived experience of chronic pain and ACEs who together are investigating the relationship between ACEs and chronic pain in adulthood.

### Participants

The Lothian Birth Cohort 1936 (LBC1936) is a longitudinal population cohort administered by the University of Edinburgh, which aims to understand the biological processes involved in cognitive, brain, and general ageing (Deary et al., 2007; Taylor et al., 2018). It is important to note that all participants were children during World War II. The cohort is derived from participants of the Scottish Mental Survey of 1947 (SMS1947), which tested the cognitive capacities of 11-year-old Scottish school children (*n =* 70,805). As described in detail elsewhere (Deary et al., 2007; Taylor et al., 2018), surviving participants of SMS1947 living in the Lothian region of Scotland were recruited and screened for eligibility for inclusion in LBC1936 at its inception in 2004 at a mean age of 69.5 ± 0.8 years (*n =* 1,091). All remaining and consenting participants have been characterised approximately every 3 years since (wave 2: 72.5 ± 0.7 years, *n =* 866; wave 3: 76.3 ± 0.7 years, *n =* 697; wave 4: 79.3 ± 0.6 years, *n =* 550; and wave 5: 82 ± 0.5 years, *n =* 431), with wave 6 taking place in 2022. In this study, our survey was mailed to surviving members of the cohort (*n =* 342) in October 2022 as a follow-up to wave 6.

### Procedures

Participant contact and data management were conducted via the LBC1936 administration team, who are solely responsible for liaising with participants, questionnaire distribution, and pseudo-anonymisation of all data entries. Participants received the study information, instructions, and consent form together with the survey (Supplementary Material). Completion and return of the survey along with a signed consent form indicated willingness to participate. All surveys returned by post to the administrative team of LBC1936 by February 2023 were included in the study. There were no additional eligibility criteria.

This study also had input from members of the CAPE patient and public involvement group, throughout the research cycle (CAPE, 2024). This group consists of eight individuals with lived experiences of ACEs and chronic pain. The group provided feedback on the research question, the participant-facing study materials, the study questionnaire, and the provision of appropriate support for participants.

### Instrument development

The questionnaire consisted of two sections: one on chronic pain and one on ACEs (Supplementary Material). Questions on chronic pain were adapted from the UK Biobank Pain Web Questionnaire (UKB-PWQ), which itself was based on well-established validated pain measurement tools such as the Brief Pain Inventory and Le Questionnaire Douleur Neuropathique 4 (DN4) (Bouhassira et al., 2005; Zelman et al., 2005; Wynick et al., 2022). The UKB-PWQ assesses the presence, duration, distribution, severity, and impact of chronic pain. There are also items relating to neuropathic pain features (taken from the DN4), other pain-related features (such as waking unrefreshed, cognition, and depression), and the use of medication to treat pain. Elements of the UKB-PWQ have been tested in people living with chronic pain and have been used in population-based studies (Meng et al., 2020; Rönnegård et al., 2022; Baskozos et al., 2023; Tanguay-Sabourin et al., 2023). The component questions align with those used by other major international consortia investigating pain, such as the DOLORisk study (Hébert et al., 2021).

Questions on ACEs were adapted from the World Health Organisation’s ACE-IQ (World Health Organization (WHO), 2018). The ACE-IQ was developed to provide a comparable measure of a broad range of ACEs (including forms of abuse, neglect, household challenges, and external challenges) across low-, middle-, and high-income countries. It has been extensively used and validated (Ho et al., 2019; Kidman et al., 2019; Pace et al., 2022; Casas-Muñoz et al., 2023), though it is designed to be administered by a trained interviewer, not to be completed by individuals directly and therefore required modification.

Some questions from the UKB-PWQ and ACE-IQ were modified in response to feedback from patient and public partners (who have lived experience of chronic pain and ACEs) and researchers from CAPE and LBC1936. The chronic pain section was considered too long and so some questions were removed (e.g., the body map of potential pain sites). Numerical rating scales were considered too abstract and so a green-yellow-red wedge scale was added to help participants visually calibrate their responses. Some of the ACE-IQ items were considered to have the potential to trigger unpleasant thoughts or memories, especially as our questionnaire would be completed by participants directly (in the absence of a trained interviewer). A similar theme regarding ACE questionnaires has previously been noted (Felitti et al., 1998; Mendel et al., 2021). Thus, we reworded some items, removed elements of significant concern (e.g., explicit references to acts of sexual assault), and provided the details of relevant UK-based organizations that could provide additional support at the end of the questionnaire. Finally, we added a free text section at the end of the questionnaire so that participants could add additional comments or provide context to their responses if they felt it appropriate to do so.

### Measures

Both sections of our questionnaire contain dichotomous (“Yes” or “No) and Likert scale response measures for severity (“No problem,” “Slight/mild problem,” “Moderate/considerable problem,” or “Severe problem”) and frequency (“Never,” “Once,” “A few times” and “Often” or “Never,” “Sometimes,” “Most of the time” and “Always”). Section one on pain additionally contained 11-point continuous numerical rating scales to rate pain intensity (0–10 with 0 representing no pain and 10 representing worst pain imaginable), pain interference (0–10 with 0 representing pain does not interfere and 10 representing pain interferes completely) and analgesia (0%–100% with 0% representing no pain relief and 100% representing complete pain relief). A full version of the survey is available in Supplementary Material. Assessment of participant responses revealed a Cronbach’s alpha of 0.79 [0.70–0.85] in section one (based on the UKB-PWQ) and 0.80 [0.76–0.84] in section two (based on the ACE-IQ) of our pain and ACE questionnaire, indicating good internal consistency (Oláh et al., 2023). The latter rating is consistent with the assessment of the original ACE-IQ in our previous study, in which the questionnaire was also assessed to have good internal consistency, reliability and criterion validity (Mosler et al., 2025).

Participants were considered to have chronic pain if they responded “Yes” to question A1 (“Are you troubled by pain or discomfort, either all the time or on and off, that has been present for more than 3 months?”) and “possible neuropathic pain” if they responded positively to ≥3 of the 7 questions from the DN4, in line with research and clinical guidelines (Finnerup et al., 2016). Pain interference was assessed across seven domains (general activity, mood, walking ability, work, relationships, sleep, and enjoyment of life), and the highest score for any item was used to establish the following categories: 0 – none, 1–3 = mild interference, 4–6 = moderate interference, 7–10 = severe interference.

The 26 ACE questions adapted from the ACE-IQ were classified into 13 ACE types within 4 ACE domains (Table 1). Participants were considered to have experienced an ACE if they responded positively (i.e., any response other than “never”) to any question, except for emotional neglect (questions D1 and D2) for which a higher threshold was set (see Supplementary Material for discussion). Therefore, the maximum ACE score across all domains was 13.

**TABLE 1:** Classification of ACE domains, ACE types, and questions from the study questionnaire.

Domain	ACE type	Questions
Abuse	Emotional	G1; G2i; G2ii; G3i
	Physical	G3ii
	Sexual	G4
Neglect	Emotional	D1; D2
	Physical	D3; D4ii; D5
Household challenges	Divorce/separation of parents or death of a parent/guardian	E3; E4
	Domestic violence	F1; F2
	Incarceration of a household member	E2
	Mental illness of a household member	E1
	Substance use by a household member	D4i
External challenges	Bullying	H1
	Community violence	H2; I1; I2; J3; J4
	Displacement	J1; J2

The study questionnaire is available in the supplementary material.

Many participants chose to enter comments in the free-text box provided at the end of the study questionnaire. These comments were redacted to remove potentially identifiable details and evaluated together. Common themes (additional pain details, physical punishment, emotional support in interpersonal relationships, ACE responses are individual, positive childhood experiences, and comments on the process) can be found in the Supplementary Material. Participants failing to respond to question A1 (*n =* 12) were excluded from statistical analyses but remained in descriptive statistics of ACE exposures.

### Statistical analysis

Data were visualised using RStudio (Version 4.4.1) and statistical comparisons performed using SPSS (Version 29.4.3). Summary data are presented as percentage (count) for categorical variables and mean (standard deviation) for continuous variables. Differences between groups were assessed using a z-test of proportions or Mann-Whitney U test, where specified. The number of ACE types experienced was categorised into the following ACE groups: 0, 1, 2, 3, and ≥4. Analyses between ACE groups and outcomes were performed using multinomial logistic regression. Regression model covariates were sex and deprivation index at age 11. The latter is established in the LBC1936 and incorporates the number of people living in the household, the number of rooms in the home, the number of people sharing toilet facilities, and whether the toilet facilities were indoor or outdoor (Johnson et al., 2011). We also performed sensitivity analyses using the number of ACE types as a continuous variable (ACE count). Sensitivity analyses between ACE count and outcomes were performed using linear regression.

Many previous studies have used a narrower range of ACEs. Therefore, we performed identical multinomial logistic regression analyses on a restricted set of responses relating to childhood maltreatment (emotional/physical/sexual abuse and emotional/physical neglect), the ACEs measured by the most commonly used questionnaires (Mosler et al., 2025). These data were derived from questions relating to domains of abuse and neglect outlined in Table 1.

### Ethical approval

Ethical approval for LBC1936 was obtained from the Multicentre Research Ethics Committee for Scotland (wave 1, MREC/01/0/56), the Lothian Research Ethics Committee (wave 2, LREC/2003/2/29), and the Scotland A Research Ethics Committee (waves 3-6, 07/MRE00/58). All participants provided written informed consent and were provided the opportunity to “prefer not to answer” to any question (Supplementary Material). The study questionnaire was reviewed and approved by the LBC Organizing Committee.

## Results

### Participant characteristics

The study questionnaire was mailed to the 342 surviving participants of LBC1936. We received 229 responses (response rate 67%), with 95% (217/229) completing Section 1 (chronic pain) and 100% (229/229) completing Section 2 (ACEs). Participant characteristics are shown in Table 2. The mean age was 86 (SD 0.3) years, and approximately half (51%, 116/229) of participants identified as female. The 113 remaining LBC1936 cohort members who did not respond had the same age and sex distribution (mean age 86 years, 50% female).

**TABLE 2:** Demographic details of LBC1936 participants who responded to the study.

Demographic item	Total	Female	Male
Number, n	229	116	113
Age, mean (SD)	86 ± 0.3	86 ± 0.3	86 ± 0.3
Deprivation index at age 11, mean z-score (range)	−0.2 (−3.0–12.0)	−0.2 (−3.0–11.0)	−0.2 (−3–12.0)
Chronic pain, n (%)			
Yes	132 (58%)	76 (66%)	56 (50%)
No	85 (37%)	31 (27%)	54 (48%)
Missing	12 (5%)	9 (8%)	3 (3%)
Chronic pain features			
Single site, n (%)	21 (16%)	14 (18%)	7 (13%)
Multi-site, n (%)	111 (84%)	62 (82%)	49 (88%)
“Possible” neuropathic pain, n (%)	20 (15%)	10 (13%)	10 (18%)
Pain score (last 24 h), mean (SD)	4.5 (1.9)	4.4 (2.0)	4.8 (1.8)
Pain lasting ≥5 years, n (%)	37 (28%)	17 (22%)	20 (36%)
Chronic pain interference, n (%)			
None	7 (5%)	5 (7%)	2 (4%)
Mild	23 (17%)	15 (20%)	8 (14%)
Moderate	46 (35%)	26 (34%)	20 (36%)
Severe	45 (34%)	23 (30%)	22 (39%)
Missing	11 (8%)	7 (9%)	4 (7%)
ACEs, n (%)			
0	41 (18%)	32 (28%)	9 (8%)
1	42 (18%)	22 (19%)	20 (18%)
2	52 (23%)	26 (22%)	26 (23%)
3	38 (17%)	16 (14%)	22 (20%)
4	14 (6%)	6 (5%)	8 (7%)
5	17 (7%)	6 (5%)	11 (10%)
6	11 (5%)	4 (3%)	7 (6%)
7	4 (2%)	0 (0%)	4 (4%)
8	8 (4%)	4 (3%)	4 (4%)
9–13	2 (1%)	0 (0%)	2 (2%)
Missing	0 (0%)	0 (0%)	0 (0%)

Deprivation at age 11 is a compound measure comprising the number of people in the household, the number of rooms in the home, the number of people sharing toilet facilities, and whether toilet facilities were indoor or outdoor (Johnson et al., 2011). ACE, adverse childhood experience; n, number; SD, standard deviation.

### Chronic pain

Survey responses revealed that 58% (132/229) of participants reported chronic pain (Table 2), of whom 52% (69/132) had lived with pain for 1–5 years and 28% (37/132) had lived with pain for >5 years. Females were more likely to report chronic pain than males (difference in proportions 20% [7%–33%], χ^2^(1, 217) = 9.2, p = 0.002). The most frequently reported location of pain was the back (54%, 71/132), though most participants (84%, 111/132) reported pain affecting multiple sites. Of those reporting chronic pain, 15% (20/132) reported possible neuropathic pain (with ≥3 features from the seven DN4 items). Participants reported their worst pain rating score in the last week as 5.1 (SD 2.3) and their average pain rating score as 4.0 (SD 2.0). When considering the impact of chronic pain, 35% (46/132) and 34% (45/132) reported moderate or severe pain interference respectively. Furthermore, 52% (68/132) of participants with chronic pain reported taking medications to treat pain.

### Adverse childhood experiences

**FIGURE 1:**
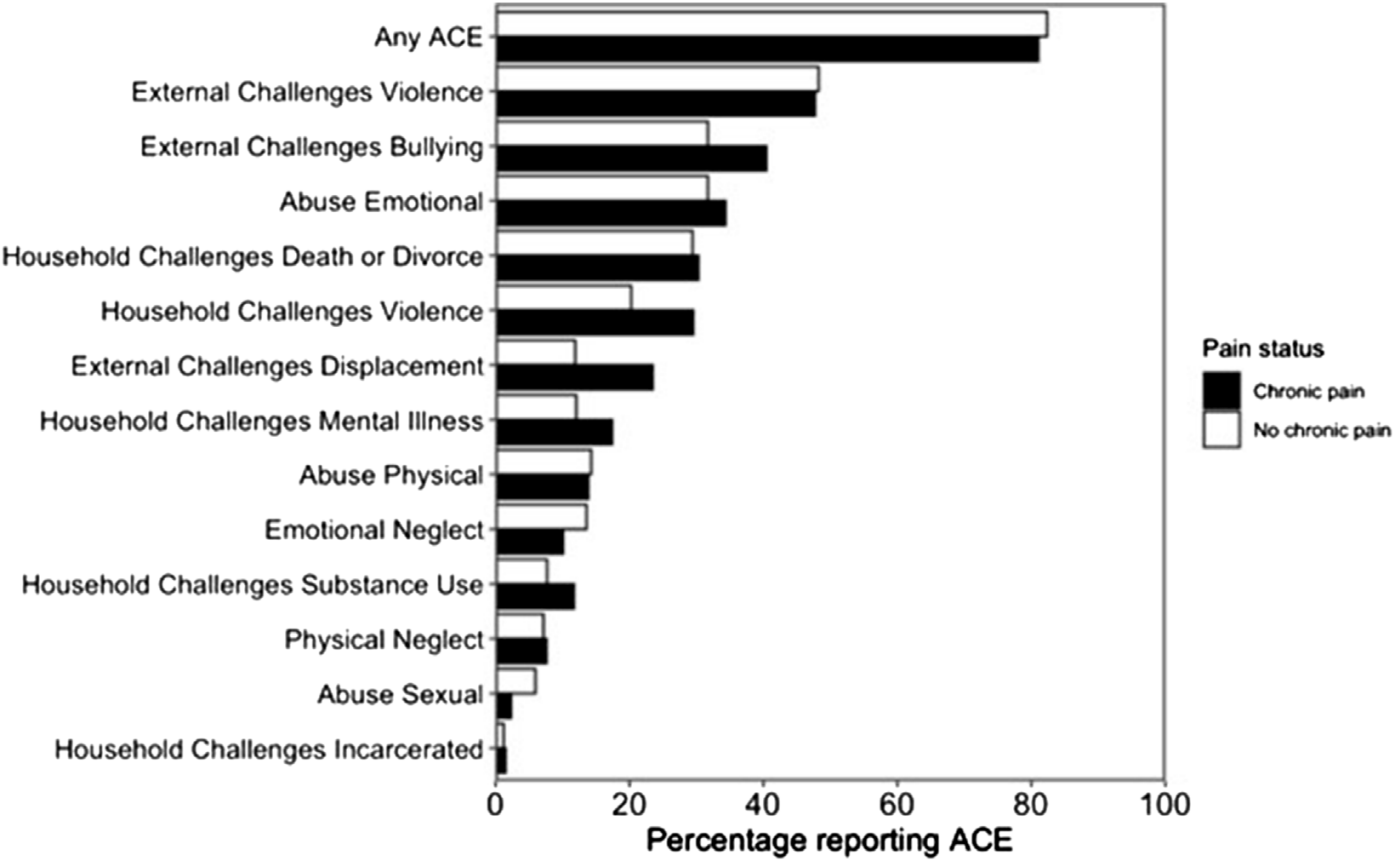
The proportion of ACEs reported by study participants. ACE, adverse childhood experience.

Most participants (82%, 188/229) reported exposure to ≥1 ACE, with 25% (56/229) reporting ≥4 (Table 2; Figure 1). The most frequently reported ACE was community violence (48%, 109/229, Table 3). Compared to females, males were significantly more likely to report exposure to any ACE (p < 0.001), physical neglect (p = 0.010), bullying (p = 0.016), and community violence (p < 0.001) (Table 3).

**TABLE 3:** ACEs reported by LBC1936 participants.

ACE domain	ACE type	Total^a^	Female^b^	Male^c^	Z-test of proportions^d^
Any ACE		188 (82%)	84 (72%)	104 (92%)	20% [10%–29%] p < 0.001
Abuse	Emotional	76 (33%)	35 (30%)	42 (37%)	7% [−5%–19%] p = 0.262
	Physical	32 (14%)	14 (12%)	17 (15%)	3% [−6%–12%] p = 0.490
	Sexual	9 (4%)	5 (4%)	4 (4%)	−1% [−6%–4%] p = 0.764
Neglect	Emotional	25 (11%)	11 (10%)	16 (14%)	4% [−4%–12%] p = 0.352
	Physical	16 (7%)	3 (3%)	14 (12%)	9% [2%–15%] p = 0.010
Household challenges	Divorce/separation of parents or death of a parent/guardian	69 (30%)	34 (29%)	36 (32%)	3% [−9%–15%] p = 0.600
	Domestic violence	57 (25%)	24 (21%)	34 (30%)	9% [−2%–20%] p = 0.118
	Incarceration of a household member	3 (1%)	2 (2%)	1 (1%)	−1% [−4%–2%] p = 0.575
	Mental illness of a household member	37 (16%)	17 (15%)	19 (17%)	2% [−8 to 11] p = 0.720
	Substance use by a household member	22 (10%)	8 (7%)	15 (13%)	6% [−2%–14%] p = 0.170
External challenges	Bullying	80 (35%)	32 (28%)	50 (44%)	15% [3%–28%] p = 0.016
	Community violence	110 (48%)	30 (26%)	79 (70%)	44% [32%–56%] p < 0.001
	Displacement	44 (19%)	19 (16%)	24 (21%)	5% [−5%–15%] p = 0.332

ACE, adverse childhood experience; CI, confidence interval.

^a^ 229 participants.

^b^ 116 participants.

^c^ 113 participants.

^d^ presented as difference [95% CI] p-value.

Recall of ACEs may be affected by cognitive symptoms (such as problems with memory, thinking skills, and/or concentration) in older adults. Therefore, we compared reporting of ACEs in those who reported cognitive symptoms with those who did not. Cognitive symptoms were reported by 57% (130/229) of participants. There was no difference in the number of ACEs reported between those who did (mean 2.8, SD 2.2) and those who did not (mean 2.2, SD 2.0) report cognitive symptoms (Mann-Whitney-U test, p = 0.101).

### Adverse childhood experiences and chronic pain

There was no observed relationship between any of the 13 individual ACEs (Figure 1) or ACE group (Table 4) and chronic pain status. There was no significant difference between ACE group and pain severity in the past 24 h (one-way ANOVA: F4, 124 = 0.44, p = 0.78). There was no association between ACE group and either pain interference or pain spread (Supplementary Material, Tables 1 and 2).

**TABLE 4:** Multinomial logistic regression analysis of ACE group and chronic pain status.

Number of ACEs	Total^a^	No chronic pain^b^	Chronic pain^c^	Odds ratio [95% CI]	P-value
0	40 (18%)	15 (18%)	25 (19%)	REF	REF
1	37 (17%)	18 (21%)	19 (14%)	0.81 [0.31 to 2.10]	0.66
2	49 (23%)	22 (26%)	27 (21%)	0.99 [0.40 to 2.40]	0.98
3	37 (17%)	13 (15%)	24 (18%)	1.70 [0.62 to 4.40]	0.32
≥4	54 (25%)	17 (20%)	37 (28%)	2.00 [0.79 to 5.20]	0.14

The analysis is adjusted for sex (female OR, 2.8 [95% CI, 1.5 to 5.1], p < 0.001) and deprivation at age 11 (OR, 1.0 [95% CI, 0.9 to 1.2], p = 0.68). ACE, adverse childhood experience; CI, confidence interval; REF, reference group.

^a^ 217 participants.

^b^ 85 participants.

^c^ 132 participants.

### Sensitivity analyses

When considering the number of ACE types as a continuous variable, we found no significant relationship between ACE count and pain severity, pain interference, or number of body sites affected by pain (Supplementary Material, Figure 1). When considering a more restricted selection of ACEs, we found no significant relationship between childhood maltreatment and chronic pain status (Supplementary Material, Table 3).

## Discussion

The LBC1936 cohort offers a rare opportunity to establish the prevalence and characteristics of pain, exposure to ACEs, and the potential association between ACEs and chronic pain in the growing but understudied “oldest old” age category (United Nations, 2015). In this study, chronic pain affected more than half of respondents, and so was more prevalent in this cohort than in the general adult population (Fayaz et al., 2016; Sá et al., 2019; Zimmer et al., 2022; Rometsch et al., 2025). Females reported a higher prevalence of chronic pain, in line with prior studies in younger age groups (Fayaz et al., 2016; Zimmer et al., 2022; Ferrari et al., 2024; Rometsch et al., 2025). A large majority of those with chronic pain reported pain affecting multiple sites, with 15% indicating chronic neuropathic pain characteristics similar to levels reported for UK Biobank participants (Baskozos et al., 2023). A third of LBC1936 participants with chronic pain indicated severe pain interference with at least one aspect of their lives. Despite this, only approximately half of those with chronic pain reported taking pain medications.

Most respondents recalled exposure to at least one ACE and a quarter of participants indicated ≥4, with males reporting more than females, in common with previous studies in working age adults (Marryat and Frank, 2019; Pace et al., 2022). Overall, the level of one or more ACE exposure (82%) corresponded to the highest levels found in prior studies using the ACE-IQ [34]. Previous studies that have assessed older populations have generally seen a lower-than-average reporting of ACEs, although these analyses were based on 6 to 9 ACE items (Rhee et al., 2019; O’Shea et al., 2021). Importantly, the entire cohort of LBC1936 lived through World War II and many reported childhood exposures to displacement and other external challenges, exposures that were not captured by most prior studies.

Despite numerous studies linking ACEs and chronic pain in adolescents and working age-adults (Groenewald et al., 2020; Tidmarsh et al., 2022; Bussières et al., 2023; Nicolson et al., 2023; Lou et al., 2024; Timmins et al., 2024) there was no association between ACEs and chronic pain in LBC1936 participants, either when considering the full list of 13 ACEs or when considering childhood maltreatment (5 ACEs) alone. There are several reasons why this might be the case. Firstly, there is a high potential for survivorship bias given that ACEs are linked to numerous long-term health conditions, multimorbidity, and early mortality (Felitti et al., 1998; Brown et al., 2009; Bellis et al., 2014; Hughes et al., 2017; Senaratne et al., 2024). Surviving LBC1936 participants may be resilient to the impact of ACE exposure on health and early mortality. Secondly, it is possible that different age groups may have different pathophysiological processes leading to chronic pain, which may in turn be influenced to different extents by prior ACE exposure. For example, one study in a community pain clinic suggested that younger adults have a greater degree of psychopathology-related pain whilst older adults have more biomedical pathology (Lakha et al., 2023). Although LBC1936 participants were in their late 80s, most reporting chronic pain had experienced it for less than 5 years. It is possible that this late-onset chronic pain is more related to natural ageing and less influenced by prior ACE exposure. It would be interesting to explore this possibility in future analysis of large population cohorts examining relationships between ACEs and chronic pain linked to age-related disease versus chronic pain with no such link.

A key strength of our study was input of people with relevant lived experiences who, alongside researchers from CAPE and the LBC1936 team, adapted the well validated UKB-PWQ and ACE-IQ for use by older people. As far as we are aware, this is the first use of a survey tool for chronic pain or ACE exposure that has been developed with overt input from people with relevant lived experiences. There are calls for the use of trauma informed care, guided in part by growing evidence of the impact of childhood trauma on a range of health outcomes including chronic pain (Oral et al., 2016; Grossman et al., 2021; Emsley et al., 2022). We argue that this approach can assist in the design of instruments that effectively balance content validity with usability, supporting their integration into trauma informed healthcare settings (Mosler et al., 2025).

### Limitations

There are also limitations to this study. Although we regard our modifications to the ACE-IQ with input from people with relevant lived experiences as an advantage, we acknowledge that this may affect comparability to the original instrument as they cannot be considered fully equivalent to the originals. In our study, ACEs were assessed retrospectively in people who have survived beyond many of their peers and so associations with chronic pain may be subject to recall and survivorship bias. The former is a common challenge for research into this area, as prospective measures of ACEs are uncommon due to ethical and practical considerations. Whilst there may be additional concerns about recall in the people in the “oldest old” age-group, the reporting of ACEs was similar between those that did and those that did not experience cognitive symptoms (including problems with memory). It may be unwise to generalise the findings in a homogeneous population of people all exposed to a common experience of World War II to others who have not experienced this exposure. It will be important for additional studies to establish whether our findings are consistent in other octogenarians.

Evolving societal norms and technological advancements will likely introduce new forms of childhood adversity that may pose challenges to the assessment of ACEs in subsequent studies of contemporary generations. For instance, children today may be exposed to stressors that were not encountered by the current oldest generations, such as cyberbullying, which could have distinct implications for long-term health outcomes, including chronic pain. It will be important to monitor emerging ACEs and consider these in future assessments to establish whether the lack of association persists as younger generations reach advanced age.

## Conclusion

A lack of an association between adverse childhood experiences and chronic pain in the people within the “oldest old” age-group has potentially important implications when considering approaches to their treatment. Whilst understanding the full context of an individual’s lived experience is an important component of the delivery of person-centred care, placing additional emphasis on the role of trauma in this age group may not be as beneficial as in younger adults. There is also evidence to suggest that pharmacological therapies may cause more harm in those with a greater ACE burden (Senaratne et al., 2025), though whether this translates to the “oldest old” age-group is also unclear and should be a focus of future research. As the global population continues to age, understanding these questions will become more important in determining how best to manage chronic pain in older adults.

## Data Availability

The raw data supporting the conclusions of this article will be made available by the authors, without undue reservation.
