# Supplementary material for: Adverse childhood experiences and chronic pain in adults aged 86: findings from the Lothian Birth Cohort 1936
Source: Front Aging. 2025 Oct 16;6:1657525. doi: 10.3389/fragi.2025.1657525 (PMC12571862; doi:10.3389/fragi.2025.1657525)
Supplement: Supplementary file 1 [file DataSheet1.docx]

Adverse childhood experiences and chronic pain in adults aged 86: findings from the Lothian Birth Cohort 1936 – Supplementary Material

Dhaneesha NS Senaratne^1,2^, Sam Singleton^3^, Kate Timmins^4^, Jeanette Spiteri^3^, Paul Redmond^5^, Adele Taylor^5^, Janie Corley^5^, Danielle Page^5^, Janine Rennie^3^, Huan Wang^2^, Madeleine Verriotis^6^, Suellen M Walker^6^, Debajit Sen^7^, Gary J Macfarlane^4^, Lesley A Colvin^1,2^, Line Caes^8^, Simon R Cox^5^, and Tim G Hales^1,3^

^1^ Chronic Pain Research Group, School of Medicine, University of Dundee, Dundee, UK

^2^ Division of Population Health & Genomics, School of Medicine, University of Dundee, Dundee, UK

^3^ Institute of Academic Anaesthesia, Division of Neuroscience, School of Medicine, University of Dundee, Dundee, UK

^4^ Aberdeen Centre for Arthritis and Musculoskeletal Health (Epidemiology Group), School of Medicine, Medical Sciences and Nutrition, University of Aberdeen, Aberdeen, UK

^5^ Lothian Birth Cohorts, Department of Psychology, University of Edinburgh, Edinburgh, UK

^6^ Great Ormond Street Institute of Child Health, University College London, London, UK

^7^ Department of Rheumatology. University College London Hospitals. NHS Foundation Trust, London, UK

^8^ Division of Psychology, Faculty of Natural Sciences, University of Stirling, Stirling, UK

[Study questionnaire 2](#_Toc198639152)

[Scoring of ACE responses 19](#_Toc198639153)

[Themes from free text comments 20](#_Toc198639154)

[Table 1 22](#_Toc198639155)

[Table 2 23](#_Toc198639156)

[Table 3 24](#_Toc198639157)

[Figure 1 25](#_Toc198639158)

**SECTION 1: QUESTIONNAIRE ON CHRONIC PAIN**

**Section A is about pain and where in your body you have experienced it.**

**A1.** Are you troubled by pain or discomfort, either all the time or on and off, that has been present **for more than 3 months**? (*If NO, please go to A5)*

| **Yes** | **No** | **Prefer not to answer** |
| --- | --- | --- |
|  |  |  |

**A2.** How long have you been suffering with this pain or discomfort?

| **3-12 months** | **1-5 years** | **More than 5 years** | **Prefer not to answer** |
| --- | --- | --- | --- |
|  |  |  |  |

**A3. Thinking about the last 24 hours of this pain**, how would you rate it on a 0-10 scale? (See the pain scale and give your pain rating in the box below).


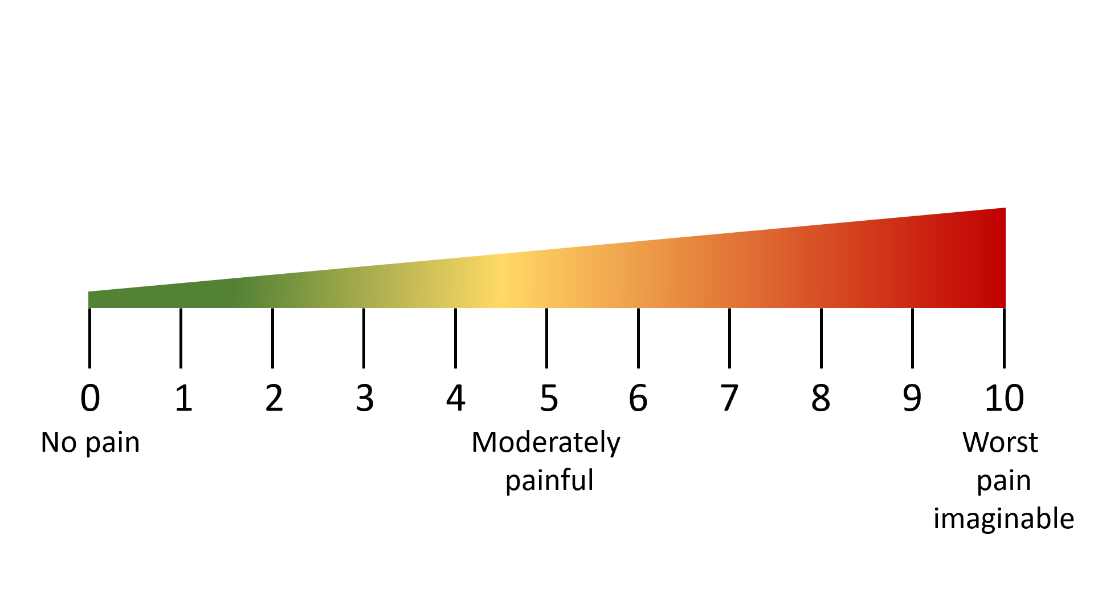


| **Pain rating (0-10):** | **Prefer not to answer** |
| --- | --- |
|  |  |

**A4.** Now we are going to ask you about where you experience this pain or discomfort**.**

**In the last 3 months** have you experienced pain or discomfort in any of the following areas?

|  | | **Yes** | **No** | **Prefer not to answer** |
| --- | --- | --- | --- | --- |
| **A4a.** | All over your body |  |  |  |
| **A4b.** | Head |  |  |  |
| **A4c.** | Face |  |  |  |
| **A4d.** | Neck or shoulders |  |  |  |
| **A4e.** | Back |  |  |  |
| **A4f.** | Stomach or Abdomen |  |  |  |
| **A4g.** | Hips |  |  |  |
| **A4h.** | Knees |  |  |  |
| **A4i.** | Arms |  |  |  |
| **A4j.** | Hands |  |  |  |
| **A4k.** | Feet |  |  |  |
| **A4l.** | Is the pain present in both your feet? |  |  |  |
| **A4m.** | Legs |  |  |  |
| **A4n.** | Chest |  |  |  |
| **A4o.** | Another part or parts of your body (please give details) |  |  |  |

**A5.** For each of the 3 symptoms that follow, indicate the level of severity experienced over the **past week**.

|  | | **No problem** | **Slight/mild problem**  generally mild or intermittent | **Moderate/considerable problem**  often present and/or at a moderate level | **Severe**  **problem**  continuous, life disturbing problems | **Prefer not to answer** |
| --- | --- | --- | --- | --- | --- | --- |
| **A5a.** | Fatigue |  |  |  |  |  |
| **A5b.** | Waking unrefreshed |  |  |  |  |  |
| **A5c.** | Cognitive symptoms. *For example, problems with memory, thinking skills and/or concentration* |  |  |  |  |  |

**A6.** For each of the 3 symptoms that follow, indicate the level of severity experienced over the **past 6 months**.

|  | | **No problem** | **Slight/mild problem**  generally mild or intermittent | **Moderate/considerable problem**  often present and/or at a moderate level | **Severe**  **problem**  continuous, life disturbing problems | **Prefer not to answer** |
| --- | --- | --- | --- | --- | --- | --- |
| **A6a.** | Pain or cramps in lower abdomen |  |  |  |  |  |
| **A6b.** | Depression |  |  |  |  |  |
| **A6c.** | Headache |  |  |  |  |  |

**Section B is about what the pain you experienced during the last week feels like.**

*If you have not experienced pain during the last week, please go to Section D.*

**B1.** Please answer this question by **thinking about the pain that bothers you most**.

Does the pain have the following characteristic(s)?

|  | | **Yes** | **No** | **Prefer not to answer** |
| --- | --- | --- | --- | --- |
| **B1a.** | Burning |  |  |  |
| **B1b.** | Painful cold |  |  |  |
| **B1c.** | Electric shocks |  |  |  |

**B2. Is the pain that bothers you most** associated with the following symptom(s) in the same body area?

|  | | **Yes** | **No** | **Prefer not to answer** |
| --- | --- | --- | --- | --- |
| **B2a.** | Tingling |  |  |  |
| **B2b.** | Pins and needles |  |  |  |
| **B2c.** | Numbness |  |  |  |
| **B2d.** | Itching |  |  |  |

**Section C**

**Now we would like to know a bit about how the pain you experienced during the last week is impacting on your life.** *If you have not experienced pain in the last week, please go to Section D.*

Please answer these questions by **thinking about the pain that bothers you most**. Use the 0 – 10 scale:


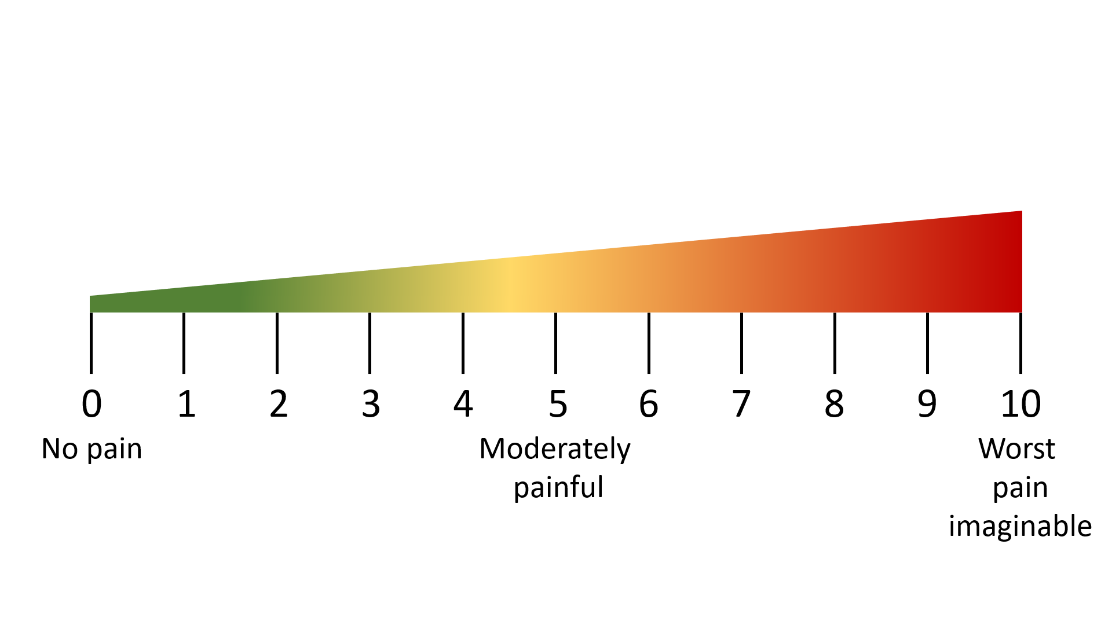


|  | | **Pain rating (0 – 10)** | **Prefer not to answer** |
| --- | --- | --- | --- |
| **C1.** | Rate your pain by selecting the number that best describes your pain at its **worst** in the **past week**. |  |  |
| **C2.** | Rate your pain by selecting the number that best describes your pain at its **least** in the **past week**. |  |  |
| **C3.** | Rate your pain by selecting the number that best describes your pain on **average**. |  |  |
| **C4.** | Rate your pain by selecting the number that best describes how much pain you have **right now**. |  |  |

**C5a.** Are you taking pain medication(s)? *(If NO, tick ‘No’ then go to C6)*

| **Yes** | **No** | **Prefer not to answer** |
| --- | --- | --- |
|  |  |  |

**C5b.** In the **past week**, how much relief have pain treatments or medications provided?

*Please use 0 - 100% scale in 10% increments:*

*
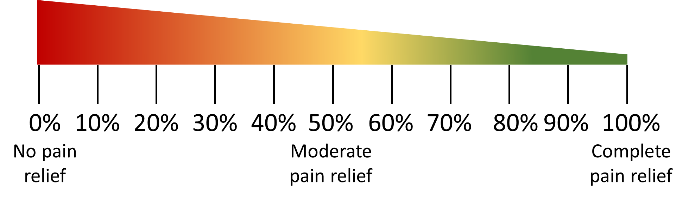
*

| **Pain rating (0-100%):** | **Prefer not to answer** |
| --- | --- |
|  |  |

**C6.** See the scale below and select the number from the scale (0 – 10) that describes how, **during the past week**, pain has interfered with your:


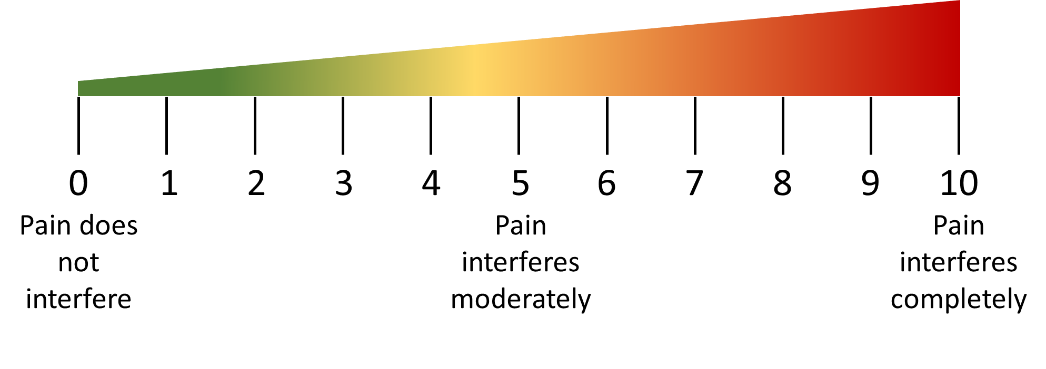


|  |  | **Pain rating (0 – 10)** | **Prefer not to answer** |
| --- | --- | --- | --- |
| **C6a.** | General Activity |  |  |
| **C6b.** | Mood |  |  |
| **C6c.** | Walking ability |  |  |
| **C6d.** | Normal work (includes both work outside the home and housework) |  |  |
| **C6e.** | Relations with other people |  |  |
| **C6f.** | Sleep |  |  |
| **C6g.** | Enjoyment of life |  |  |

**SECTION 2: QUESTIONNAIRE ON STRESSFUL EVENTS IN CHILDHOOD**

**Section D**

**These questions are about exposure to difficult experiences when you were a child** and may bring back traumatic memories. If you have concerns about any of the issues covered in the questionnaire, please find a list of sources of support at the end of this questionnaire.

We would now like to ask about your relationship with your parents or guardians. When you were growing up, **during the first 18 years of your life**:

|  | | **Never** | **Sometimes** | **Most of the time** | **Always** | **Prefer not to answer** |
| --- | --- | --- | --- | --- | --- | --- |
| **D1.** | Did your parent(s)/guardian(s) fail to listen to your problems and worries? |  |  |  |  |  |
| **D2.** | Did your parent(s)/guardian(s) take an interest in what you were doing with your free time when you were not at school or work? |  |  |  |  |  |

|  | | **Never** | **Sometimes** | **Most of the time** | **Always** | **Prefer not to answer** |
| --- | --- | --- | --- | --- | --- | --- |
| **D3.** | How frequently did your parent(s)/guardian(s) fail to provide food when they had the means to do so? |  |  |  |  |  |
| **D4i.** | How frequently did your parent(s)/guardian(s) use alcohol or drugs in a worrying way?  *(If never, please go to D5)* |  |  |  |  |  |

|  | | **Never** | **Sometimes** | **Most of the time** | **Always** | **Prefer not to answer** |
| --- | --- | --- | --- | --- | --- | --- |
| **D4ii.** | Did use by your parent(s)/guardian(s) of alcohol or drugs result in you being neglected? |  |  |  |  |  |
| **D5.** | How often did your parent(s)/guardian(s) fail to send you to school even when it was available? |  |  |  |  |  |

**Section E**

These questions are about your **family environment** when you were a child, **during the first 18 years of your life**:

|  | | **Yes** | **No** | **Prefer not to answer** |
| --- | --- | --- | --- | --- |
| **E1.** | Did you live with a parent, guardian or household member who experienced depression, anxiety or other mental health problem? |  |  |  |
| **E2.** | Did you live with a parent, guardian or household member who was sent to prison? |  |  |  |
| **E3.** | Did your parents separate or divorce? |  |  |  |
| **E4.** | Did your mother, father or guardian die? |  |  |  |

**Section F**

These questions are about **things you may have heard or seen in your home when you were a child.** These are things that may have been done to another household member but not necessarily to you.

When you were growing up, **during the first 18 years of your life:**

|  | | **Never** | **Once** | **A few times** | **Often** | **Prefer not to answer** |
| --- | --- | --- | --- | --- | --- | --- |
| **F1.** | Did you see or hear a parent, guardian or household member in your home being shouted at, insulted or humiliated? |  |  |  |  |  |

|  | | **Never** | **Once** | **A few times** | **Often** | **Prefer not to answer** |
| --- | --- | --- | --- | --- | --- | --- |
| **F2.** | Did you see or hear a parent, guardian or household member in your home being physically abused? |  |  |  |  |  |

**Section G**

These questions are about **certain very difficult things that you may have experienced** when you were a child.

When you were growing up, **during the first 18 years of your life:**

|  | | **Never** | **Once** | **A few times** | **Often** | **Prefer not to answer** |
| --- | --- | --- | --- | --- | --- | --- |
| **G1.** | Did a parent, guardian or other household member shout at you, insult or humiliate you? |  |  |  |  |  |
| **G2i.** | Did a parent, guardian or other household member **threaten to** abandon you (e.g., by leaving you or making you leave your home)? |  |  |  |  |  |
| **G2ii.** | Did a parent, guardian or other household member abandon you (e.g., by leaving you or making you leave your home)? |  |  |  |  |  |
| **G3i.** | Did a parent, guardian or other household member **threaten to** physically abuse you? |  |  |  |  |  |

|  | | **Never** | **Once** | **A few times** | **Often** | **Prefer not to answer** |
| --- | --- | --- | --- | --- | --- | --- |
| **G3ii.** | Did a parent, guardian or other household member physically abuse you? |  |  |  |  |  |
| **G4.** | Did someone ually abuse you? |  |  |  |  |  |

**Section H**

These questions are about certain things when you were growing up that may have happened **outside your household such as being bullied.** Bullying, in this context, is when a person or group of people say or do bad and unpleasant things to another person. It is also bullying when a person is teased a lot in an unpleasant way or when a person is left out of things on purpose.

When you were growing up, **during the first 18 years of your life:**

|  | | **Never** | **Once** | **A few times** | **Often** | **Prefer not to answer** |
| --- | --- | --- | --- | --- | --- | --- |
| **H1.** | How often were you bullied? |  |  |  |  |  |

**The next question is about physical fights that occur when two young people of about the same strength or power choose to fight each other.**

When you were growing up, **during the first 18 years of your life:**

|  | | **Never** | **Once** | **A few times** | **Often** | **Prefer not to answer** |
| --- | --- | --- | --- | --- | --- | --- |
| **H2.** | How often were you in a physical fight? |  |  |  |  |  |

**Section I**

The next questions are about how often, when you were a child, **you may have witnessed certain things in your neighbourhood or community.**

When you were growing up, **during the first 18 years of your life:**

|  | | **Never** | **Once** | **A few times** | **Often** | **Prefer not to answer** |
| --- | --- | --- | --- | --- | --- | --- |
| **I1.** | Did you witness actual violent attack in the community (e.g., assault, stabbing, shooting). |  |  |  |  |  |
| **I2.** | Did you witness credible threats of community violence (e.g., with a weapon such as a knife or a gun). |  |  |  |  |  |

**Section J**

The next questions are about whether you did or did not **experience any of the following events when you were a child.** The events are all to do with collective violence, including wars (such as World War 2), terrorism, political or ethnic conflicts, genocide, repression, disappearances, torture and organized violent crime such as banditry and gang warfare.

When you were growing up, **during the first 18 years of your life:**

|  | | **Yes** | **No** | **Prefer not to answer** |
| --- | --- | --- | --- | --- |
| **J1.** | Were you forced to go and live in another place due to any of these events? |  |  |  |
| **J2.** | Did you experience the deliberate destruction of your home due to any of these events? |  |  |  |
| **J3.** | Were you beaten up by soldiers, police, militia, or gangs? |  |  |  |

|  | | **Yes** | **No** | **Prefer not to answer** |
| --- | --- | --- | --- | --- |
| **J4.** | Was a family member or friend killed or beaten up by soldiers, police, militia, or gangs? |  |  |  |

**Many thanks for taking the time to answer these questions**. We really appreciate your contribution. Your answers will be used, along with other Lothian Birth Cohort data, to better understand chronic pain.

**If you wish to tell us anything else relevant to this questionnaire, then please use the space below.**

**Some of the questions in this questionnaire may have brought back traumatic memories. If this is the case and you would like support, please consider contacting the following organisations**:

**Wellbeing Scotland,** a voluntary organisation with services across Scotland. The organisation provides a wide range of holistic services for individuals and families whose life experiences have impacted negatively on their wellbeing. Support from Wellbeing Scotland is available Monday – Friday 9 am to 5 pm. You can get in touch by phone (01324 630 100) or by email: [info@wellbeingscotland.org](mailto:info@wellbeingscotland.org), or by contacting Janine Rennie who represents people with lived experiences of pain as lead of the Chronic Pain Advisory Group ([janine@wellbeingscotland.org](mailto:janine@wellbeingscotland.org)).More information can be found on the website: <https://www.wellbeingscotland.org/>

**MIND** is a charity that provides free advice and support for mental health. MIND has an information line, that can be called for information and advice on support services. This information line is open Monday-Friday 9 am to 6 pm. MIND can be contacted on 0300 123 3393. Importantly, MIND has resources for those suffering mental health crises: <https://www.mind.org.uk/need-urgent-help/>

**Breathing Space** is a free, confidential, phone and webchat service for anyone in Scotland over the age of 16 experiencing low mood, depression or anxiety. Breathing Space offers support Monday – Thursday 6 pm to 2 am and 24 hours over weekends Friday – Sunday. The Breathing Space number is free to call on 0800 838587. More information can be found on the website: <https://breathingspace.scot/>

**The Samaritans** provides emotional support for anyone who's struggling to cope, who needs someone to listen without judgment or pressure. The Samaritans are available to people across the UK, 24 hours every day. The Samaritans are available by phone (116 123) or by email [jo@samaritans.org](mailto:jo@samaritans.org). More information can be found on the website: <https://www.samaritans.org>

**HAVOCA – Help for Adult Victims of Child Abuse** is run by survivors for adult survivors of child abuse and provides support, friendship and advice for any adult whose life has been affected by childhood abuse. The website offers support groups, forums, blogs and advice on finding a therapist: <https://www.havoca.org/>

**Victim Focus** is an organisation dedicated to challenging poor practice, discrimination, and oppression of victims of abuse, trauma, and violence. Victim Focus carries out a lot of research on improving services for victims and has a range of resources on its website, including a list of councillors across the UK: <https://victimfocus.org.uk/directory-of-practitioners/>

# Scoring of ACE responses

The potential range of responses to different ACE questions were either “never”/“sometimes”/“most of the "time”/“always”, “never”/“once”/“a few times”/“often”, or “no”/“yes”. ACEs were coded into binary outcomes, so a response of “never” or “no” was taken to mean a negative response and all other affirmatory responses were taken to mean a positive response. The exception was questions relating to emotional neglect: D1 “Did your parent(s)/guardian(s) fail to listen to your problems and worries?” and D2 “Did your parent(s)/guardian(s) take an interest in what you were doing with your free time when you were not at school or work?”. The intermediate responses to these questions (“sometimes”/“most of the time”) were deemed by researchers from CAPE and LBC1936 and some members of the patient and public involvement group to not reach sufficient threshold for emotional neglect. However, this was not universally accepted, with one person with lived experience commenting: “Having a parent not listening to you, or taking no interest, even some of the time is crushing as a child, which then has a knock-on effect in relationships as we get older”.

# Themes from free text comments

Almost half of participants (49%, 112/229) chose to provide additional feedback in the free text box at the end of the PACE-Q. The following repetitive themes were highlighted:

## Additional pain details

The PACE-Q asks about pain duration, features, and interference but does not ask about the causes or treatment options. Many participants chose to provide these additional details; for example, one participant stated that *“My chronic pain is the result of an unsuccessfully treated attack of shingles which leaves me with constant pain in the face and head.”* Some participants also provided additional insight into the impact of their pain: *“The pain I have in my back is due to osteoporosis. This means when I do anything for about half an hour I have to sit down. I am very frustrated at this.”*

## Physical punishment

Some participants reflected that definitions of physical abuse have changed over the years. One stated that: *“A belting in the distant past for being naughty would today be regarded as physical abuse – I regarded that as well-earned. No harm done. But well remembered.”* Many participants agreed that some forms of physical punishment for misbehaviour were socially acceptable during their childhood but would be considered differently now. However, not all participants were unphased by this normalisation of physical punishment: *“I remember a small girl being caned again and again by another teacher. That frightened me and I remember it now.”* Illustrating the common practice of physical punishment at the time the LBC1936 participants were children, some participants reported physical punishment in response to poor academic performance: *“Frequently got belted by teacher for poor spelling and writing”*; and even in hospital: *“I spent 6 weeks in hospital when I was 7 years old. I was regularly smacked by a nurse for wetting the bed, she definitely bullied me. This was a very stressful time.”*

## Emotional support in interpersonal relationships

Some participants identified a lack of emotional connection between individuals in their household, most commonly between parents: *“I never thought of them as a unit, but as 2 separate individuals…I don't think either of my parents really understood or sympathised with the needs of the other”*. Others reported that their parents failed to support them when other adults interacted with them in a negative or potentially harmful way: *“My grandmother was the power in our family…I felt she was hard on me and I felt abandoned by my mother who did not defend me.”*

## ACE responses are individual

It was common to find opposing interpretations of similar reported events; as one participant commented: *“Stress is a very subjective thing.”* The members of the LBC1936 were all children during World War II and have overlapping war experiences. Some reported lifelong effects: *“I can still remember the extreme fear I felt at the sound of the sirens and then noise of planes overhead. As an adult I have always suffered from anxiety relative to being away from home. Maybe there is a connection.”* Others seemed relatively unaffected: *“We lived for part of the war in the countryside under the Germany-London flight path. There were often bombs falling all round us and sometimes anti-aircraft guns in our lane. But we never worried about it.”*

## Positive childhood experiences

Many participants were keen to highlight that their childhood conditions were positive: *“I appreciate I had a very privileged childhood living in a loving family.”* Some were keen to emphasise this, even if they felt that they had suffered negative events: *“My father died in 1944 during active service. My mother as a widow brought my brother and I up in an exemplary manner. I had an excellent childhood despite losing my father."*

## Comments on the process:

A few participants commented on the process of completing the questionnaire. One stated: *“I found this questionnaire difficult to answer especially part two. Attitudes, beliefs, lifestyle etc were so different from now…We were learning life’s lessons not expecting a garden of roses.”* Another asked: *“(Rhetorical) question from me - Is research based on impressions and memories from 70 years ago reliable?”*

# Table 1

Table 1: Multinomial logistic regression analysis of ACE group and pain interference.

| **Number of ACEs** | **Total** | **Low pain interference** | **High pain interference** | **Odds ratio** [95% CI] | **P-value** |
| --- | --- | --- | --- | --- | --- |
| 0 | 22 (18%) | 6 (20%) | 16 (18%) | REF | REF |
| 1 | 17 (14%) | 7 (23%) | 10 (11%) | 0.48 [0.12 to 1.88] | 0.29 |
| 2 | 27 (22%) | 5 (17%) | 22 (24%) | 1.51 [0.39 to 5.92] | 0.55 |
| 3 | 22 (18%) | 8 (27%) | 14 (15%) | 0.53 [0.14 to 2.03] | 0.35 |
| ≥4 | 33 (27%) | 4 (13%) | 29 (32%) | 2.09 [0.48 to 9.06] | 0.32 |

Note. Analysis is adjusted for sex (female OR = 1.70 [0.65 to 4.38], p = 0.28) and deprivation at age 11 (OR = 1.05 [0.87 to 1.27], p = 0.61). ACE: adverse childhood experience, CI: confidence interval, REF: reference group. ^a^121 participants, ^b^30 participants, ^c^91 participants.

# Table 2

Table 2: Multinomial logistic regression analysis of ACE group and pain spread.

| **Number of ACEs** | **Total** | **Single site** | **Multiple sites** | **Odds ratio** [95% CI] | **P-value** |
| --- | --- | --- | --- | --- | --- |
| 0 | 25 (40%) | 5 (50%) | 20 (38%) | REF | REF |
| ≥4 | 37 (60%) | 5 (50%) | 32 (62%) | 3.03 [0.50 to 18.29] | 0.23 |

Note. Analysis is adjusted for sex (female OR = 0.23 [0.04 to 1.37], p = 0.11) and age 11 deprivation (OR = 0.88 [0.69 to 1.12], p = 0.28). ACE: adverse childhood experience, CI: confidence interval, REF: reference group. ^a^62 participants, ^b^10 participants, ^c^52 participants.

# Table 3

Table 3: Multinomial logistic regression analysis of childhood maltreatment group and chronic pain status.

| **Number of ACEs** | **Total** | **No chronic pain** | **Chronic pain** | **Odds ratio** [95% CI] | **P-value** |
| --- | --- | --- | --- | --- | --- |
| 0 | 127 (59%) | 49 (58%) | 78 (59%) | REF | REF |
| 1 | 48 (22%) | 18 (21%) | 30 (23%) | 1.20 [0.58 to 2.40] | 0.65 |
| 2 | 29 (13%) | 13 (15%) | 16 (12%) | 0.80 [0.34 to 1.80] | 0.59 |
| 3 | 9 (4%) | 4 (5%) | 5 (4%) | 1.00 [0.24 to 4.10] | 0.98 |
| ≥4 | 4 (2%) | 1 (1%) | 3 (2%) | 1.90 [0.17 to 20.0] | 0.61 |

Note. Analysis is adjusted for sex (female OR = 2.4 [1.4 to 4.3], p = 0.002) and age 11 deprivation (OR = 1.03 [0.91 to 1.2], p = 0.63). ACE: adverse childhood experience, CI: confidence interval, REF: reference group. ^a^217 participants, ^b^85 participants, ^c^132 participants.

# Figure 1

Figure 1. Correlation analysis of ACE count and pain outcomes in participants reporting chronic pain (n = 132). **A)** From left to right, pain severity in the past 24 hours (R^2^ < 0.01; p = 0.69), average severity in the past week (R^2^< 0.01; p > 0.99), lowest severity in the past week (R^2^< 0.01; p > 0.99), and highest in the past week (R^2^= 0.02; p > 0.74). **B)** From top left to bottom right, pain interference with enjoyment of life (R^2^= 0.02; p = 1), general activity (R^2^= 0.02; p = 0.86), mood (R^2^= 0.04; p = 0.21), normal work/studying (R^2^< 0.01; p = 1), relations with other people (R^2^= 0.03; p = 0.39), sleep (R^2^= 0.05; p = 0.13) and walking ability (R^2^= 0.13; p = 0.1). **C)** Number of pain sites affected (R^2^= 0.03; p = 0.05).


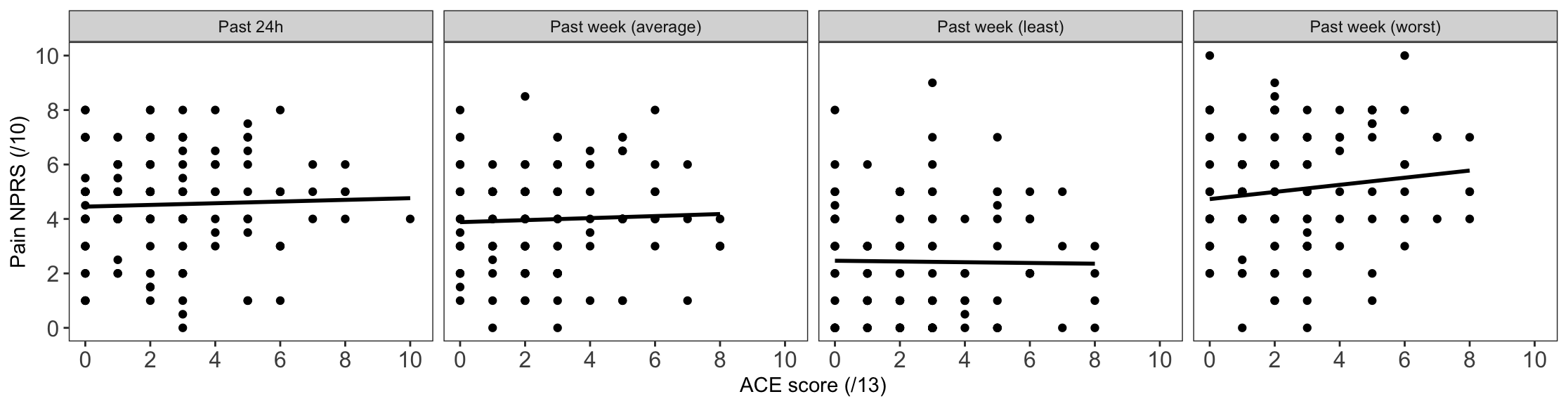


A

B

C


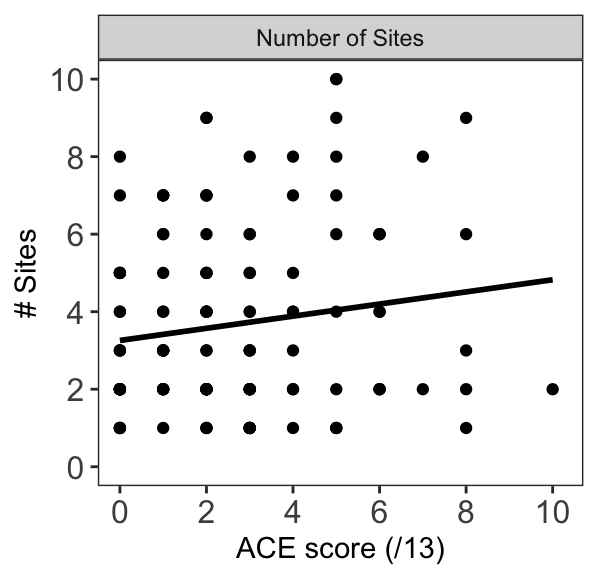

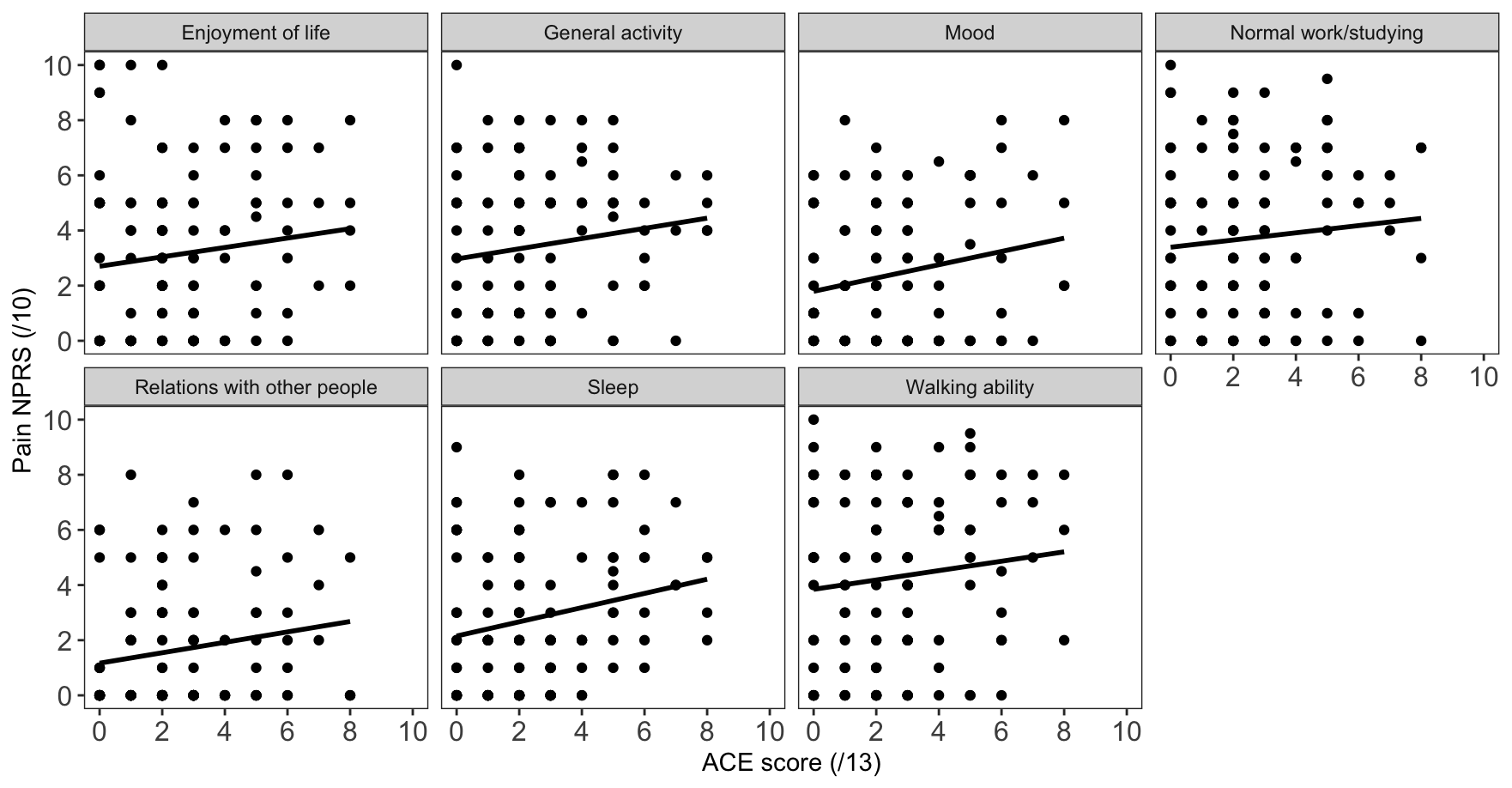


Pain NRS

Pain interference NRS

Pain sites

ACE count

ACE count

ACE count
